# Exploring thematic dimensions of breast pumping discussions in an online community using large language models and multiple correspondence analysis

**DOI:** 10.3389/fnut.2026.1800363

**Published:** 2026-03-30

**Authors:** Efstathios Kaloudis, Justin E. Silpe, Eleni Mourelatou, Karla Damian-Medina, Vasiliki Bountziouka

**Affiliations:** 1 Computer Simulations, Genomics and Data Analysis Lab, Department of Food Science and Nutrition, University of the Aegean, Myrina, Lemnos, Greece; 2 PumpKin Baby Inc., Princeton, NJ, United States; 3 Population, Policy and Practice Department, GOS Institute of Child Health, University College London, London, United Kingdom; 4 Department of Cardiovascular Sciences and Department of Population Health Sciences, School of Healthcare, University of Leicester, Leicester, United Kingdom

**Keywords:** breast pumping, breastmilk, exclusive pumping, large language models, natural language processing, Reddit, sensory properties, social media

## Abstract

**Background:**

Breast pumping is a central part of infant feeding for many parents, yet professional guidance often gives limited attention to psychosocial and experiential aspects. Online communities offer valuable insight into how parents describe and navigate these experiences. Large language models provide new opportunities to analyze large volumes of online discussions.

**Objective:**

To identify and describe major thematic patterns in online discussions about breast pumping and infant feeding, with a focus on sensory concerns related to stored milk and their links to parental distress.

**Methods:**

Four subreddits related to pumping and breastfeeding were selected, and 107,447 relevant posts, extracted from November 2010 to December 2024, were retained in the analysis. Relevant content was identified using a combination of keyword matching and semantic similarity. Eleven binary-coded questions covering practical, experiential, and psychosocial topics were developed. Llama 3.1 8B was used for systematic classification. Ten questions achieving classification accuracy above 75% were retained. Multiple correspondence analysis was used to explore underlying thematic dimensions. A Supplementary keyword co-occurrence analysis examined discussions of sensory changes in stored milk.

**Results:**

Four main dimensions were identified, explaining 52.5% of total variance. Dimensions 1 (16.2%) and 2 (13.6%) differentiated discussions focused on feeding decisions and emotional experiences from those explicitly addressing sensory changes in expressed milk. Dimensions 3 (11.8%) and 4 (10.9%) distinguished concerns about perceived effects of pumping on milk quality from practical and work-related aspects of pumping. In *r/ExclusivelyPumping*, frozen milk storage was discussed in 7.8% of posts, while 2.7% mentioned sensory concerns (taste, smell, soapy, metallic, sour). Among posts referencing lipase, 43.0% also discussed frozen milk stash, and 21.1% mentioned scalding as a preventive strategy.

**Conclusion:**

These findings suggest gaps in current guidance on milk storage and quality and highlight the need for more targeted support and interventions for pumping parents. Although sensory changes in stored breastmilk were mentioned relatively infrequently, they emerged as a distinct theme in pumping-related discussions. The frequent co-occurrence of lipase concerns with frozen milk stashes reflects a recurring situation in which parents discover sensory changes only after substantial effort has been invested in building a stored milk supply.

## 1 Introduction

Breastfeeding is widely recognized as the optimal form of infant nutrition, providing essential nutrients, immunological protection, and developmental benefits (1). The World Health Organization recommends exclusive breastfeeding for the first 6 months of life, followed by continued breastfeeding alongside complementary foods up to 2 years or beyond (1, 2). However, direct breastfeeding is not always feasible due to factors such as premature birth, infant hospitalization, maternal medical conditions, latch difficulties, or return to employment (3, 4). In these circumstances, breast pumping (i.e., the expression of milk using manual or electric devices) enables parents to provide breastmilk when nursing at the breast is not possible. Exclusive pumping (EP), in which all breastmilk is expressed via pump, has become an increasingly common feeding modality (5–8), yet remains underrepresented in clinical guidance and research compared with direct breastfeeding (9–11).

Parents who exclusively pump face distinct practical and psychosocial challenges, including substantial time demands, equipment and storage logistics, concerns about maintaining milk supply, and physical discomfort (4, 9, 11, 12). Psychosocial burdens are also prominent, with many EP parents reporting feelings of isolation, guilt, or inadequacy when pumping is perceived as inferior to direct breastfeeding (9, 12). Emotional wellbeing, social support, and workplace accommodation play important roles in feeding continuation and parental satisfaction, but these dimensions are often insufficiently addressed in healthcare settings (13–15).

A defining feature of pumping is the ability to store expressed milk for later use. Many parents build frozen milk “stashes” to support work schedules, increase flexibility, or provide security against supply disruptions (16, 17). However, biochemical changes during storage, and in particular lipase-mediated fat breakdown, can alter milk’s sensory properties, producing soapy, metallic, or sour tastes and odors (18–21). These changes may lead to infant rejection of stored milk, resulting in wasted milk and considerable emotional distress for parents who have invested significant effort in building a frozen supply (20, 22, 23). Although scalding milk after expression can inactivate lipase and prevent sensory changes, this practice is not widely known and adds complexity to pumping routines (24, 25). The prevalence of sensory concerns and their broader implications for pumping experiences remain poorly characterized.

Online communities have become important sources of peer support for parents navigating infant feeding decisions (12, 26, 27). Reddit, a social media platform organized into topic-specific communities, hosts several active subreddits dedicated to breastfeeding and pumping. Particularly, *r/ExclusivelyPumping* has grown into a large, dedicated space where parents share practical advice, emotional experiences, and challenges related to pumping. Such platforms provide researchers with access to large-scale, naturalistic discussions that capture parental experiences outside clinical or research settings, offering valuable insights that complement traditional qualitative methods (28, 29). However, the volume and complexity of social media data pose substantial analytical challenges.

Large language models (LLMs) offer new opportunities for analyzing large textual datasets in health research. These models can interpret context, colloquial language, and nuanced meanings, enabling scalable classification of social media content with high consistency (30, 31). Compared with keyword-based or manual coding approaches, LLM-based classification allows efficient and reproducible analysis of extensive corpora while retaining semantic depth (32). Nonetheless, limitations remain, particularly for questions requiring inference from implicit or ambiguous content. While a growing body of work has examined social media as a source of breastfeeding support, including systematic reviews of online group use (27) and qualitative studies of support communities (12), very few studies have applied large-scale computational methods to social media discussions about breast pumping specifically. A recent content analysis of *r/breastfeeding* identified themes of breastfeeding challenges, weaning, and returning to work (33), but focused on general breastfeeding rather than pumping-specific experiences. Similarly, Rosenbaum and McAlister (4) noted in their integrative review that women who exclusively express breast milk frequently report receiving more help from informal social media groups than from healthcare providers, underscoring the importance of understanding what is discussed in these communities. To our knowledge, no prior study has combined large language model-based classification with multivariate methods such as multiple correspondence analysis to systematically examine the thematic structure of pumping-related discussions at this scale. The present study therefore aimed to: (1) characterize the thematic structure of breast pumping discussions in Reddit communities using LLM-based classification and multiple correspondence analysis; (2) examine how sensory concerns, practical feeding decisions, and emotional experiences relate to one another within these discussions; and (3) evaluate the feasibility and limitations of LLM-based classification for analyzing large-scale social media data on infant feeding practices.

## 2 Materials and methods

### 2.1 Data source and collection

Reddit was selected as the primary data source due to its large, diverse user community and the depth of user-generated content covering breastfeeding and pumping topics (34). Among numerous breastfeeding-related subreddits (Supplementary Table 1), four subreddits namely *r/breastfeeding, r/breastfeedingsupport, r/ExclusivelyPumping, r/HumansPumpingMilk* were selected for this analysis as they represent the most focused community for pumping-specific discussions, with established membership since 2010 (Table 1).

**TABLE 1:** Breastfeeding-related subreddits on Reddit retained in the analysis.

Subreddit	Members (till December 2024)	Established	Focus
r/Breastfeeding	∼185K	November, 2009	General breastfeeding support
r/ExclusivelyPumping	∼55K	October, 2015	Exclusive and partial breast pumping support
r/BreastfeedingSupport	∼20K	July, 2018	Positive breastfeeding encouragement
r/HumansPumpingMilk	∼13K	April, 2021	Pumping support community

Data were obtained from publicly available archives from the subreddit *r/pushshift*, a comprehensive source of Reddit data (35). The dataset comprised anonymized content including original posts (submissions) from all four relevant subreddits from their inception through December 31, 2024. We focused on original submissions rather than comments, as submissions tend to be more structured and self-contained, making them suitable for systematic classification. For each post, the title and body text fields were extracted and concatenated for analysis. All data were stored in an SQLite database for efficient processing.

### 2.2 Posts identification and selection

A hybrid filtering approach was developed to identify posts relevant to breastfeeding and pumping, combining keyword matching with semantic similarity analysis. This two-stage approach ensured both high precision (through keyword matching) and high recall (through semantic similarity) when classifying posts in the informal, diverse linguistic environment of Reddit.

In the first stage, posts were checked against a comprehensive list of 311 breastfeeding- and pumping-related seed terms loaded from an external file. These terms included equipment names (e.g., “spectra,” “medela,” “elvie”), physiological challenges (e.g., “mastitis,” “clogged duct,” “engorgement”), emotional descriptors (e.g., “overwhelmed,” “guilt,” “anxiety”), and infant feeding behaviors (e.g., “bottle refusal,” “latch”). Posts containing any seed term via case-insensitive substring matching were immediately classified as related.

Posts without keyword matches proceeded to semantic similarity evaluation using the all-MiniLM-L6-v2 model from the Sentence-Transformers library. This model converts text into dense vector representations (embeddings) enabling comparison of semantic meaning. Eight thematic seed clusters were defined, each represented by curated seed phrases:

1. Breastfeeding: milk, boob, breastfeeding, lactation, sluck, ounce, oz, feeding, preservation
2. Pumping behavior: pumping, exclusive pumping, power pump, triple feeding, milk supply, stash, output
3. Storage and equipment: milk bags, freezer, frozen, bottle, hands-free bra, spectra, medela, sterilize, cooler, wearable pump, preservation
4. Nutrition and quality: nutrients, nutritional value, fat content, protein content, macronutrient, nutrient retention, nutrient loss, oxidation, milk quality, spoilage prevention, shelf-life, nutrient preservation
5. Medical challenges: mastitis, clogged duct, nipple pain, engorgement, letdown, milk blister, thrush, reglan
6. Emotional and mental: postpartum depression (ppd), anxiety, overwhelmed, discouraged, guilt, proud, mental health, support, lonely
7. Feeding response: bottle refusal, feeding reaction, baby gagging, combo feeding, acceptance, preference, latch
8. Safety concerns: safe to use, milk contamination, pump hygiene, sterilization, storage, milk spoilage, temperature, cleaning

Posts were classified as related if their embedding achieved cosine similarity of 0.30–0.35 or higher with at least one cluster centroid.

### 2.3 Questions development and LLM classification

Eleven binary-coded research questions were developed through iterative refinement to capture practical, experiential, and psychosocial aspects of breast pumping discussions (Table 2). Questions underwent multiple revision cycles (May through August 2025) to optimize classification accuracy and reduce LLM assumptions. Key refinements included requiring explicit mentions rather than inferences, adding clarifying instructions to prevent over-interpretation, and standardizing response options.

**TABLE 2:** Research questions used for large-language model classification.

A/A	Question	Options for answer	Achieved accuracy
1	Is a clear and explicit reason for pumping stated in the post? Select Yes only if the reason is directly stated in a clear sentence. Do not select Yes if the reason is implied, guessed from context, or based on general knowledge.	Yes/No	78%
2	Is the pumping frequency clearly and explicitly stated in the post?	Yes/No	86.8%
3	Is a pump or equipment mentioned?	Yes/No	90.4%
4	Does the post explicitly describe or compare different feeding methods? Only select Yes if the post clearly compares or switches between methods.	Yes/No	80%
5	If different feeding methods are discussed, are changes in the taste of milk mentioned?	No mention/Yes/No	100%
6	If different feeding methods are discussed, are changes in the texture of milk mentioned?	No mention/Yes/No	99.2%
7	Is infant acceptance of the milk mentioned?	Yes/No	22.8%
8	Are emotional or mental health impacts mentioned?	Yes/No	98.4%
9	Do they mention breastfeeding practices or pumping after returning to work?	Yes/No	85.6%
10	Are there any foods or supplements mentioned to increase milk supply?	Yes/No	98%
11	If foods or supplements are mentioned to increase milk supply, are any effects of these foods or supplements on milk or infant health reported?	Yes/No	100%

The final questions covered: reasons for pumping; pumping frequency; pump equipment; milk storage methods and duration; challenges and concerns; feeding methods and infant response; changes in milk taste; changes in milk texture; infant acceptance of milk; emotional and mental health impacts; work-related pumping; foods and supplements for milk supply; digital tools and apps; and sources of support.

Post classification was performed using the Llama 3.1 8B language model. Each post was independently evaluated against each research question by prompting the model with the full post content and the corresponding question, producing binary (Yes/No) or categorical responses as predefined. A consistent prompting framework was applied across all classifications, explicitly instructing the model to rely on direct textual evidence and to avoid inference from ambiguous or implied information.

Classification performance was assessed by comparing LLM outputs with parallel classifications generated using ChatGPT-4*o* on a randomly selected subset of posts. Questions were retained for analysis only if classification accuracy stabilized at > 75%. One question addressing infant acceptance of expressed milk (“Is infant acceptance of the milk mentioned?”) demonstrated poor performance (approximately 20% agreement) and was therefore excluded from subsequent analyses.

### 2.4 Statistical analysis

The ten binary classification variables for each post, with > 75% accuracy, were treated as nominal variables with two categories [Yes/No (incl. no mention)] and were used to explore latent structures in the binary LLM-classified responses, using multiple correspondence analysis (MCA). MCA is a multivariate exploratory technique for categorical data that identifies patterns of association among variable categories by projecting them into a low-dimensional space, analogous to principal component analysis for continuous variables (36).

MCA decomposed the total inertia into a set of orthogonal dimensions, ordered according to the proportion of inertia explained. The number of retained dimensions was guided by the scree plot and the cumulative proportion of inertia explained. Dimension loadings and variable contributions were examined to aid interpretation of the underlying dimensions. Category coordinates were visualized in two-dimensional plots to assess associations and co-occurrence patterns among content categories.

The “FactoMineR” (37) package was used to compute MCA, and the “factoextra” (38) package to extract and visualize the results. Analyses were performed in R 4.5.2 (39).

#### 2.4.1 Complementary keyword analysis

To complement MCA and to explore sensory-related concerns in greater depth, a targeted keyword co-occurrence analysis was conducted within *r/ExclusivelyPumping*. This subreddit was selected *a priori* due to its thematic focus on expressed milk storage and handling, and its substantially higher volume of storage- and pumping-related discussions compared with other communities. Equivalent analysis was not performed for other subreddits due to insufficient thematic density of sensory-related content, which would have limited interpretability and statistical robustness.

Specific analyses focused on: (1) the prevalence of sensory-related terms (e.g., taste, smell, soapy, metallic, sour, lipase); (2) co-occurrence between lipase-related mentions and stash- or storage-related discussions; and (3) co-occurrence of sensory concerns with indicators of emotional distress. In addition, word frequency analysis was performed on the complete *r/ExclusivelyPumping* corpus to identify the most commonly occurring terms related to milk storage, sensory properties, and infant feeding.

## 3 Results

### 3.1 Dataset characteristics

From the subreddits’ inception dates through December 2024, a total of 201,875 original posts were extracted. Following content screening, 53.3% (*n* = 107,632) were identified as relevant to breastfeeding and pumping and considered for the analysis. After excluding 185 posts that failed to meet the similarity threshold, 107,447 posts (99.8% of screened content) remained in the analysis (Supplementary Figure 1). Most relevant posts originated from *r/breastfeeding* (57%), followed by *r/ExclusivelyPumping* (36%), with equal proportions (3.3% each) from *r/HumansPumpingMilk* and *r/breastfeedingsupport*.

Across the study period (2010–2024), all four subreddits exhibited increasing engagement, although with distinct temporal trajectories (Supplementary Figure 2). Posting activity in *r/breastfeeding* showed steady growth from its inception, with a more pronounced acceleration after 2019. Between 2010–2019 and 2020–2024, the average annual number of posts increased from 1,776 to 8,816, representing an approximately 5-fold increase. In contrast, pumping-focused communities displayed later but substantially sharper increases in activity, particularly from 2020 onwards, coinciding with the COVID-19 period. For *r/ExclusivelyPumping* activity was minimal prior to 2020 (13 posts on average during 2010-2019, but rose dramatically thereafter reaching an average of 7,730 posts annually between 2020 and 2024. Similarly, *r/HumansPumpingMilk* was created in 2021 and therefore does not allow for a pre-post 2020 comparison. However, since its inception, the subreddit has shown sustained engagement averageing 701 posts through 2024, with some year-to-year fluctuation.

**FIGURE 1:**
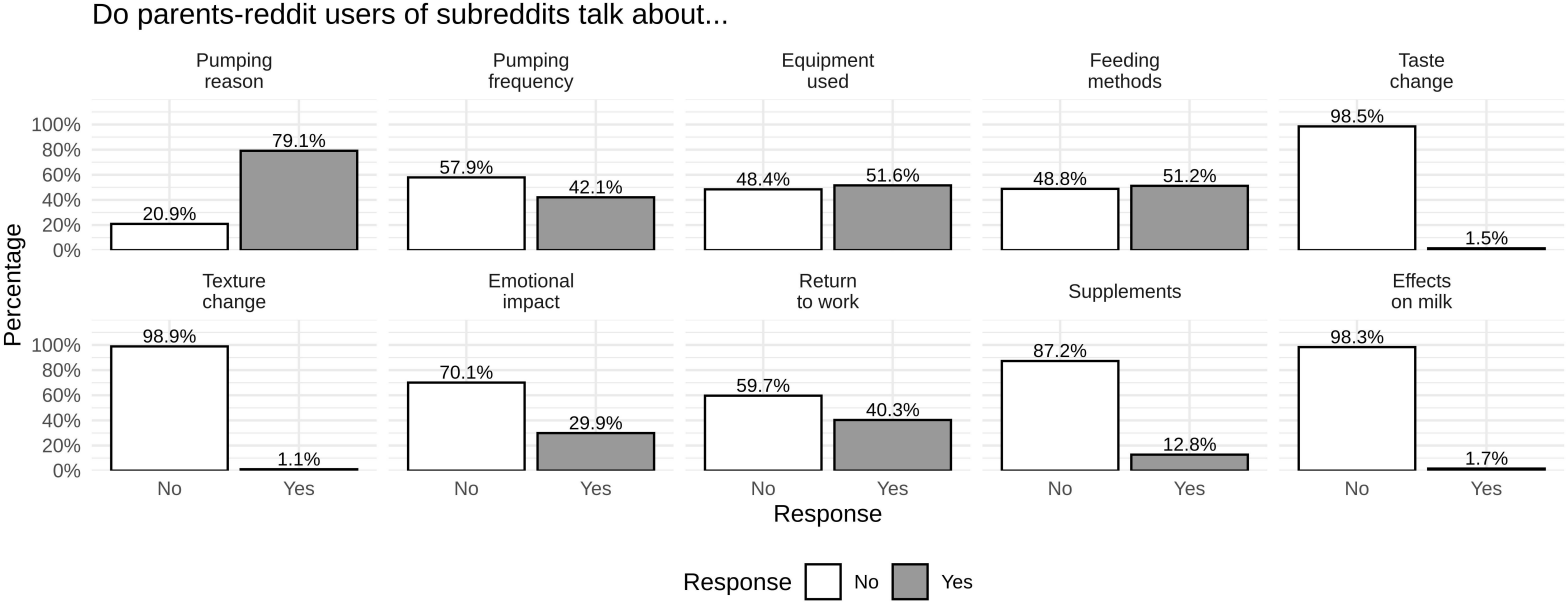
Distribution of topic mentions across pumping-related discussion categories.

Overall, pumping-related discussions were primarily focused on practical aspects, with most posts mentioning reasons for pumping (79.1%) and approximately half addressing equipment use (51.6%) and feeding methods (51.2%). Emotional (29.9%) and work-related considerations (40.3%) were less frequent but still considerable. By contrast, sensory concerns and perceived effects on milk quality were explicitly mentioned in only a small minority of posts, suggesting that these issues, while potentially impactful, are less commonly articulated in online discussions (Figure 1). Across subreddits, the distribution of topic mentions was largely consistent, with discussions most frequently addressing reasons for pumping, equipment use, feeding methods, work-related considerations, and emotional impact. Sensory-related concerns (taste, texture, and perceived effects on milk) were rare across all communities, appearing in fewer than 2% of posts regardless of subreddit (Supplementary Figure 3).

### 3.2 Occurring dimensions

MCA of the ten binary classification variables identified four principal dimensions (Supplementary Figure 4), together explaining approximately 52.5% of the total inertia in the classified content (Table 3). Dimensions were interpreted based on categories with the highest contributions (Supplementary Table 2).

**TABLE 3:** Multiple correspondence analysis dimensions.

Dimension	Inertia (%)	Cumulative (%)	Primary content
1st	16.2	16.2	Feeding practices, supplementation, and emotional engagement
2nd	13.6	29.8	Sensory properties of expressed milk
3rd	11.8	41.6	Milk quality, supplementation, and perceived effects on milk
4th	10.9	52.5	Pumping logistics, equipment, and return-to-work practices

**FIGURE 2:**
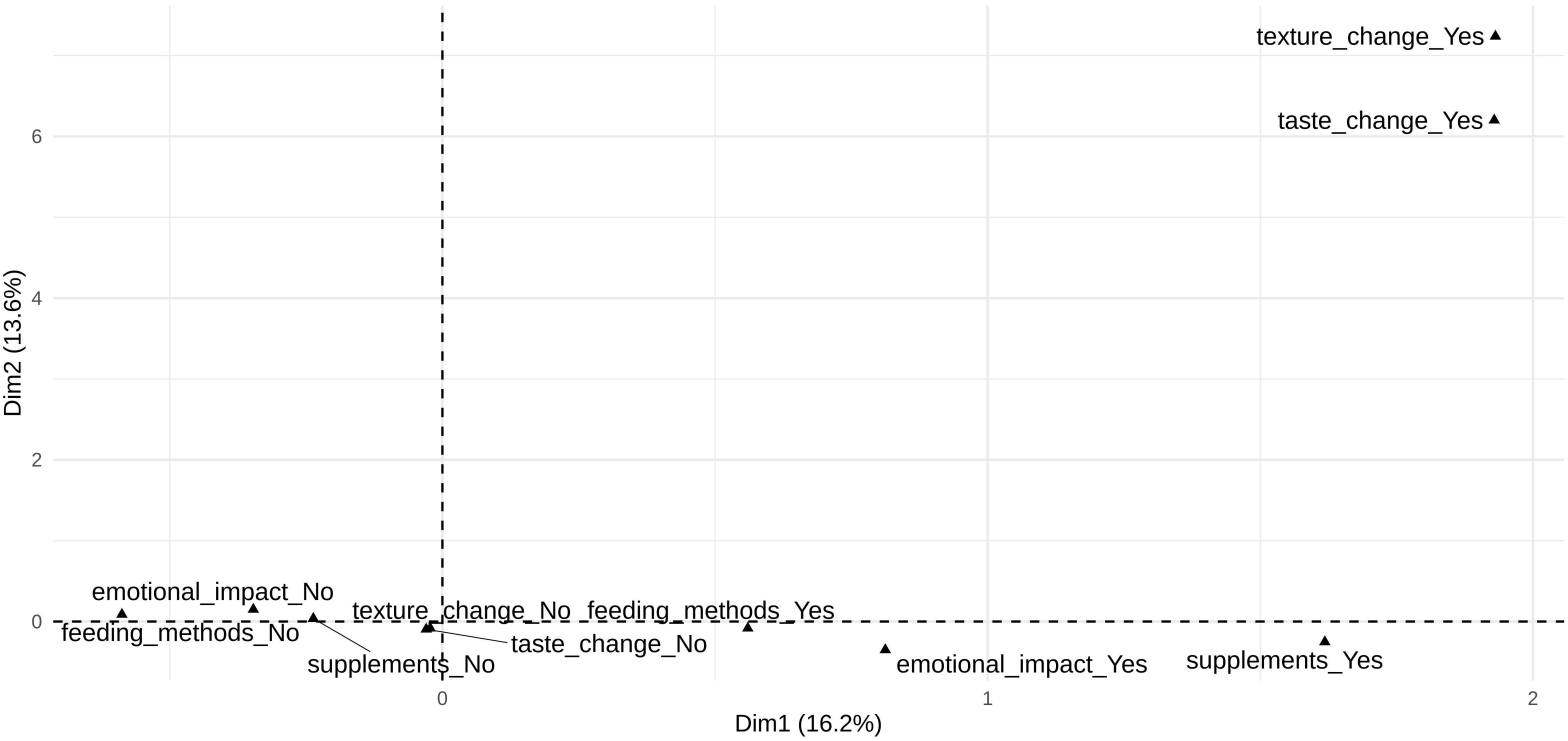
Key content categories from all subreddit posts (*n* = 107,447) contributing to the first and second dimensions of the multiple correspondence analysis. Key content categories contributing to the first two dimensions of the multiple correspondence analysis based on all subreddit posts (*n* = 107,447). Only variable categories heavily load (i.e., cos^2^ > 0.30) on the displayed dimensions are shown. Dimension 1 (16.2% of total inertia) reflects feeding practices, supplementation, and emotional engagement; Dimension 2 (13.6%) reflects sensory properties of expressed milk.

The first dimension (16.2% of total inertia) was primarily driven by categories related to feeding methods, supplement use, perceived effects on milk, and emotional impact, with particularly high contributions from *feeding methods (Yes/No)*, *supplements (Yes)*, *effects on milk (Yes)*, and *emotional impact (Yes)*. This dimension distinguishes posts that explicitly engage with decisions around infant feeding (e.g., pumped milk versus direct breastfeeding or formula), dietary or supplemental strategies intended to influence milk supply or quality, and the emotional experiences accompanying these decisions, from posts that do not explicitly reference these aspects. The second dimension (13.6%) was dominated by sensory-related categories, with *taste change (Yes)* and *texture change (Yes)* accounting for the largest contributions by a wide margin. The first two dimensions differentiate emotionally and behaviorally engaged discussions from posts focused on sensory experiences related to expressed milk, highlighting complementary but largely independent thematic structures in pumping-related discourse (Figure 2).

**FIGURE 3:**
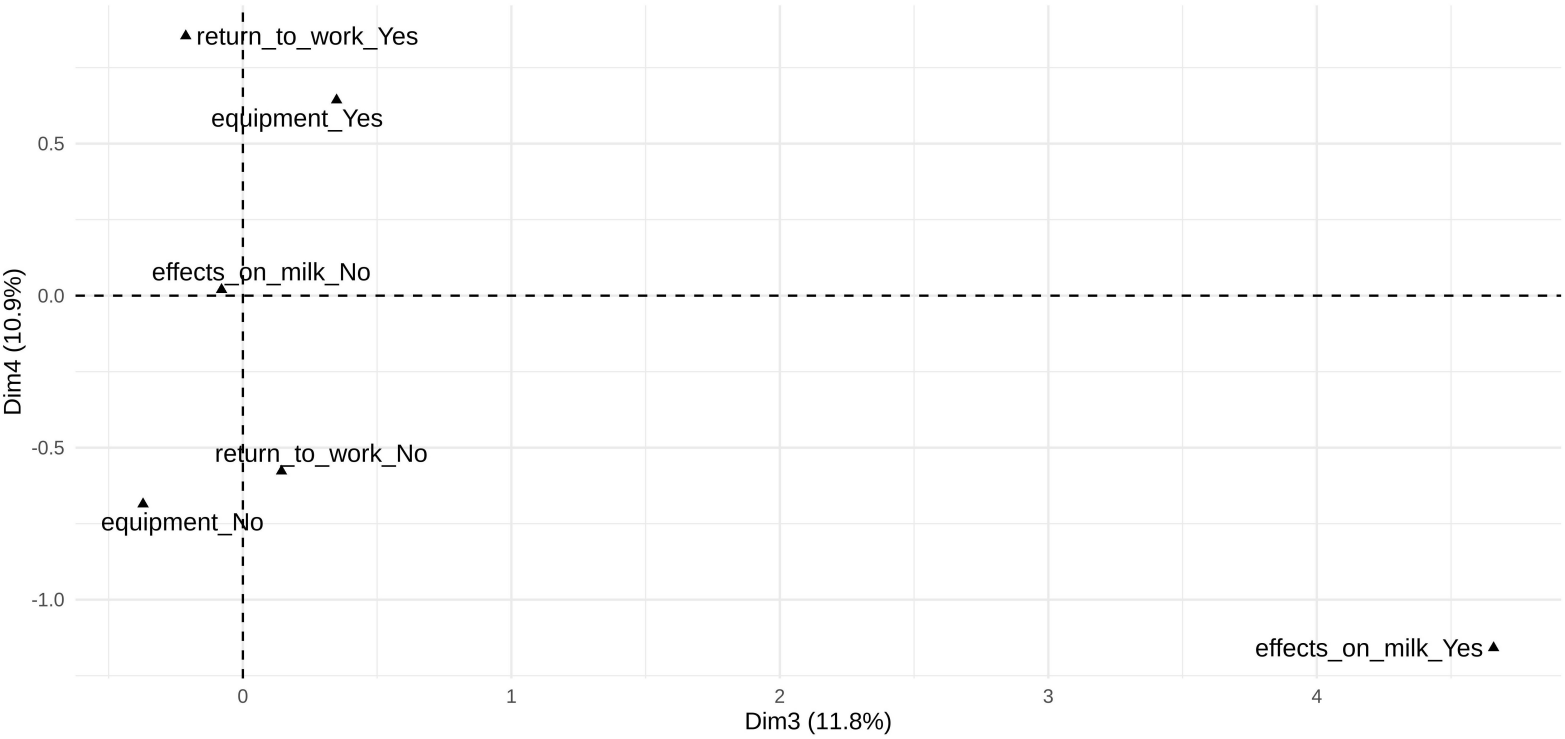
Key content categories from all subreddit posts (*n* = 107,447) contributing to the third and fourth dimensions of the multiple correspondence analysis. Key content categories contributing to the first two dimensions of the multiple correspondence analysis based on all subreddit posts (*n* = 107,447). Only variable categories heavily load (i.e., cos^2^ > 0.30) on the displayed dimensions are shown. Dimension 3 (11.8% of total inertia) reflects milk quality, supplementation, and perceived effects on milk; Dimension 4 (10.9%) reflects pumping logistics, equipment, and return-to-work practices.

The third dimension (11.8%) was characterized by high contributions from effects on *milk (Yes)*, *supplements (Yes)*, and *feeding methods (Yes/No)*, indicating a thematic focus on perceived milk quality and modification. Posts loading on this dimension commonly discussed how pumping practices, diet, or supplementation may influence milk composition, supply, or suitability for infant feeding. The fourth dimension (10.9%) was strongly driven by *equipment (Yes/No)* and *return to work (Yes/No)* categories, capturing discussions centered on the practical logistics of pumping, including pump selection and use, and the integration of pumping into work routines. The orthogonal separation between the third and fourth dimensions indicates that concerns about milk quality are largely independent of logistical or workplace considerations (Figure 3).

### 3.3 Key findings of complementary keyword analysis

Given the thematic concentration of storage- and pumping-related discussions in *r/ExclusivelyPumping*, Supplementary keyword analyses were restricted to this subreddit to enable more detailed examination of sensory concerns. Storage-related terms were among the most frequent in the corpus (Table 4), indicating that management of frozen milk reserves is a central topic within this community. Sensory-related terms (e.g., taste, smell, soapy, metallic, sour, lipase) appeared less frequently but were nonetheless present in a substantial subset of posts.

**TABLE 4:** Prevalence of storage and sensory-related terms in the corpus (*n* = 38,742).

Term	Posts containing the term (%)
Storage	
Freezer	3,472 (9.0)
Stash	3,040 (7.8)
Sensory	
Any term	1,030 (2.7)
Taste	391 (1.0)
Smell	371 (1.0)
Soapy	110 (0.3)
Sour	93 (0.2)
Metallic	65 (0.2)
Lipase	384 (1.0)

Co-occurrence analysis revealed that sensory concerns were often identified in the context of milk storage. Nearly half of posts (163/384) mentioning lipase also referenced a frozen milk stash, suggesting that sensory changes are commonly detected when stored milk is thawed for use. Discussions of mitigation strategies, such as scalding (81 posts), and concerns about wasting or discarding expressed milk (56 posts) were also evident. Importantly, sensory-related discussions frequently co-occurred with expressions of emotional distress (e.g., “devastated,” “heartbroken,” “upset,” “crying,” “sad,” 95 posts).

## 4 Discussion

This study examined large volumes of online discussions from breastfeeding- and pumping-related communities to better understand conversations about exclusive pumping. Although sensory concerns and perceived effects on milk quality were mentioned in only a small proportion of posts, the analysis showed that these issues form a clearly distinct theme within pumping-related discussions. Activity patterns also differed across communities, with pumping-focused subreddits showing later but sharper increases in participation, particularly after 2020, suggesting shifts in where and how parents seek information and support. The accelerated growth of pumping-focused subreddits from approximately 2020 onward may partly reflect the disruption of in-person lactation support during the COVID-19 pandemic, which shifted many parents toward online platforms for breastfeeding assistance (40, 41). Across discussions, practical feeding decisions were closely linked with emotional experiences. Posts about feeding methods and supplementation often included references to stress, worry, or emotional strain, indicating that pumping choices are rarely discussed as purely technical matters. At the same time, equipment use and return-to-work issues clustered together as a separate topic, reflecting the practical and structural challenges of maintaining pumping alongside employment.

The complementary keyword analysis helps clarify why these concerns may be particularly distressing. Mentions of lipase frequently co-occurred with discussions of frozen milk stashes, indicating that sensory changes are often discovered only after parents have invested considerable time and effort into building a stored supply. Pumping regularly, storing milk over long periods, and planning ahead for work or emergencies require sustained commitment. Discovering that this accumulated milk has developed off-flavors therefore represents not only a practical loss, but also an emotional one. The relatively low proportion of posts mentioning scalding as a preventive strategy suggests that awareness of available interventions remains limited within the community. Concerns about discarding milk and the co-occurrence of sensory discussions with expressions of distress further point to the emotional weight of these experiences.

These patterns are consistent with what is known about the biochemistry of breastmilk storage. Lipase activity can continue during refrigeration and freezing, leading to the release of free fatty acids that produce soapy, metallic, or rancid tastes and odors (18–21). Variation in lipase activity across individuals means that some parents never encounter this issue, while others may experience sensory changes within hours of expression (18, 42). Although scalding can inactivate lipase and prevent these changes, it adds complexity to an already demanding routine and may affect certain bioactive components of milk (24, 25, 43). These factors help explain why sensory concerns emerge as a distinct and emotionally charged theme, even if they are not widely discussed.

The low classification accuracy for infant milk acceptance further highlights how parents communicate about feeding challenges. Unlike equipment use or pumping frequency, acceptance concerns are rarely stated directly. Instead, parents tend to describe infant behavior, such as fussing, refusing a bottle, or not finishing feeds, without explicitly linking these observations to milk quality or acceptance. This may reflect uncertainty about the cause of feeding difficulties, as well as reluctance to attribute problems to expressed milk that required significant effort to produce. These findings suggest that both research tools and clinical conversations may need to focus more on behavioral descriptions rather than direct questions about acceptance.

Consistent with previous qualitative work, practical constraints such as equipment use, time demands, and emotional strain featured prominently across discussions (9–12). The emotional and psychosocial dimensions observed in our data are consistent with broader findings on the experiences of exclusively pumping parents, who frequently report feelings of isolation, stigma, and a perceived lack of support from healthcare providers (12, 44). Workplace barriers, including insufficient break time, inadequate facilities, and lack of employer understanding, have been identified as major factors shaping pumping decisions and duration (13, 15, 45). The limited clinical guidance available to exclusively pumping parents has also been noted as a significant gap (4, 10). A recent content analysis of *r/breastfeeding* by Yates et al. (33) identified similar themes, including breastfeeding challenges, returning to work, and normalizing infant behavior, supporting the consistency of patterns we observed across pumping-specific communities. Results from the MCA extends this understanding by showing how these concerns cluster into distinct but related dimensions. Equipment use and return-to-work considerations formed a clear logistical axis, highlighting the structural challenges of sustaining pumping alongside employment. In contrast, concerns about milk quality and sensory changes were largely independent of workplace or equipment issues, reinforcing the idea that these experiences represent a separate set of challenges rather than downstream consequences of logistical barriers.

From a methodological perspective, the LLM-based classification approach performed well for questions based on explicit content, supporting its use for large-scale analysis of online discussions. In contrast, the poor performance of the milk acceptance question highlights an important limitation of automated approaches when content is implicit or context dependent. During validation, several patterns emerged that help explain these strengths and limitations. Classification accuracy tended to decrease as post length increased, likely because longer posts often contain multiple topics, narrative digressions, or ambiguous information that complicate clear categorization. Accuracy was also lower for posts using more specialized or complex terminology, suggesting that domain-specific language can challenge model interpretation.

In addition, many posts did not state key details explicitly, such as pumping frequency or timing, which reduced classification performance for questions requiring precise information. This limitation was particularly evident for infant milk acceptance: most posts did not clearly indicate whether the infant accepted the milk, yet the model often inferred a positive response in the absence of explicit refusal. Similarly, when posts contained only brief mentions of keywords without sufficient context, the model tended to treat these mentions as affirmative evidence, leading to misclassification. Finally, many posts did not clearly articulate a reason for pumping; in such cases, the model frequently defaulted to classifying pumping as a matter of personal preference, reflecting a tendency toward implicit assumptions when explicit rationale was absent. This suggest that LLM-based classification performs best when information is stated clearly and explicitly, and that questions relying on inference, implicit meaning, or contextual interpretation remain challenging for automated approaches. Future work may therefore benefit from combining automated classification with targeted qualitative analysis to better capture these subtler aspects of feeding experiences.

Several additional limitations should be considered when interpreting these findings. As Reddit does not provide verified demographic information, we were unable to determine the gender or parental role of users contributing posts. However, the content and language of posts were consistent with first-person accounts by individuals who were personally lactating and pumping. We use the term “parents” inclusively throughout this paper to reflect a range of identities and the intended audience of the subreddits rather than confirmed characteristics of individual contributors. Moreover, Reddit users are not representative of all pumping parents, as participation reflects a self-selected group that may differ in experiences, resources, and concerns. The binary classification approach necessarily simplifies complex and nuanced discussions, and the focus on specific subreddits may not capture the full range of pumping-related experiences expressed in other online or offline contexts. In addition, keyword co-occurrence analysis cannot establish causal relationships, although the temporal sequence implied in stash building and subsequent sensory discovery supports the proposed interpretation.

Exclusive pumping involves practical and logistical demands, but it is also shaped by emotional experiences that are often overlooked in formal guidance and support. The patterns observed in this study highlight several opportunities to improve support for pumping parents. Healthcare providers may need to address sensory changes in stored milk more proactively, including discussion of lipase activity, strategies for testing milk acceptance early, and the potential role of scalding. Clinical assessments of feeding difficulties may also benefit from greater attention to infant behaviors, rather than relying solely on direct questions about milk acceptance. Finally, the prominence of return-to-work discussions underscores the need for workplace policies and guidance that extend beyond protected time and private space, offering practical support for equipment choice and pumping routines that can be sustained alongside employment.

## 5 Conclusion

Using large-scale online discussions, this study identified four main thematic dimensions that reflect how parents describe and make sense of pumping. Sensory changes in expressed milk emerged as a distinct theme, and complementary analyses highlighted the “stash paradox,” in which parents invest considerable effort in building frozen milk supplies only to discover sensory changes when the milk is later used. The largely implicit way in which concerns about milk acceptance are expressed suggests that these issues may be missed in both research and clinical practice. Overall, the findings point to the need for more comprehensive support for pumping parents, with greater attention to milk storage, sensory changes, and strategies that help preserve milk quality over time.

## Data Availability

The raw data supporting the conclusions of this article will be made available by the authors, without undue reservation.
