# Supplementary material for: Exploring thematic dimensions of breast pumping discussions in an online community using large language models and multiple correspondence analysis
Source: Front Nutr. 2026 Mar 30;13:1800363. doi: 10.3389/fnut.2026.1800363 (PMC13071425; doi:10.3389/fnut.2026.1800363)
Supplement: Supplementary file 1 [file Supplementary_file_1.pdf]

# *Supplementary Material*

## 1 Supplementary Figures and Tables

### 1.1 Supplementary Tables

**Supplementary Table 1:** Number of posts (or submissions, or entries) per subreddit

| Subreddit | Established | Number of Posts |
|---|---|---|
| r/AttachmentParenting | July, 2010 | 12,509 |
| r/BabyLedWeaning | January, 2014 | 13,958 |
| r/BeyondTheBump | April, 2012 | 326,142 |
| r/BoobsAndBottles | *couldn't verify* | N/A |
| **r/Breastfeeding** | November, 2009 | **193,120** |
| **r/BreastfeedingSupport** | July, 2018 | **8,831** |
| r/BreastPumps | *couldn't verify* | N/A |
| r/Daddit | August, 2010 | 209,129 |
| **r/ExclusivelyPumping** | October, 2015 | **41,752** |
| r/FormulaFeeders | June, 2010 | 25,308 |
| **r/HumansPumpingMilk** | April, 2021 | **6,209** |
| r/Mommit | September, 2010 | 166,539 |
| r/NewParents | July, 2011 | 144,582 |
| r/NurseAllTheBabies | January, 2018 | N/A |
| r/Parenting | April, 2010 | 501,003 |
| r/WorkingMoms | October, 2013 | 42,370 |
| **Total** | | **1,691,452** |

Subreddits in bold indicate the selected subreddits for the analysis.

**Supplementary Table 2:** Contributions (%) of each category to the first five dimensions

| | **Dimensions** | | | | |
|---|---|---|---|---|---|
| | **1st** | **2nd** | **3rd** | **4th** | **5th** |
| **Post mentions** | | | | | |
| Reason for pumping | | | | | |
| No | 6.67 | 1.83 | 6.25 | 0.06 | **25.8** |
| Yes | 1.77 | 0.48 | 1.65 | 0.02 | 6.83 |
| Frequency of pumping | | | | | |
| No | 2.68 | 0.80 | 0.25 | 2.82 | **23.6** |
| Yes | 3.69 | 1.10 | 0.34 | 3.88 | **32.5** |
| Equipment used | | | | | |
| No | 0.00 | 0.13 | 5.65 | **20.8** | 5.08 |
| Yes | 0.00 | 0.13 | 5.31 | **19.5** | 4.76 |
| Feeding methods | | | | | |
| No | **10.4** | 0.28 | 8.36 | 0.44 | 0.10 |
| Yes | 9.90 | 0.26 | 7.97 | 0.42 | 0.09 |
| Milk taste change | | | | | |
| No | 0.05 | 0.66 | 0.02 | 0.01 | 0.00 |
| Yes | 3.47 | **42.9** | 1.56 | 0.72 | 0.01 |
| Milk texture change | | | | | |
| No | 0.03 | 0.51 | 0.01 | 0.02 | 0.00 |
| Yes | 2.62 | **44.1** | 0.52 | 1.37 | 0.00 |
| Emotional impact | | | | | |
| No | 5.19 | 1.16 | 2.07 | 0.50 | 0.29 |
| Yes | **12.2** | 2.71 | 4.85 | 1.18 | 0.68 |
| Return to work | | | | | |
| No | 0.72 | 0.81 | 1.04 | **18.2** | 0.01 |
| Yes | 1.06 | 1.20 | 1.54 | **26.9** | 0.02 |
| Supplement intake | | | | | |
| No | 3.01 | 0.09 | 2.73 | 0.12 | 0.01 |
| Yes | **20.6** | 0.60 | **18.6** | 0.85 | 0.07 |
| Effects on milk | | | | | |
| No | 0.27 | 0.00 | 0.52 | 0.04 | 0.00 |
| Yes | **15.7** | 0.26 | **30.7** | 2.06 | 0.11 |

**Bold** fonts indicate contributions above 10%.

### 1.2 Supplementary Figures

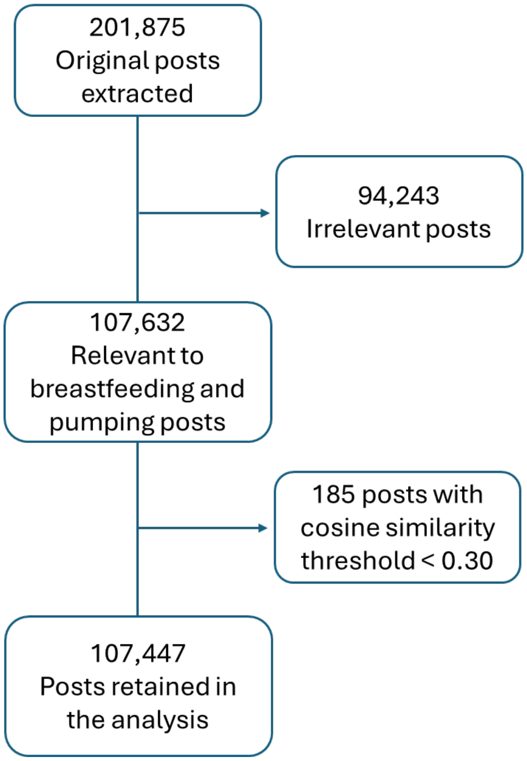


**Supplementary Figure 1.** Flowchart of data extraction and screening process.

**Legend:** From the subreddits' inception dates through December 2024, a total of 201,875 original posts were extracted. Following content screening, 53.3% (n = 107,632) were identified as relevant to breastfeeding and pumping and considered for the analysis. After excluding 185 posts that failed to meet the similarity threshold, 107,447 posts with cosine similarity threshold ≥ 0.30 (99.8% of screened content) retained for the analysis.

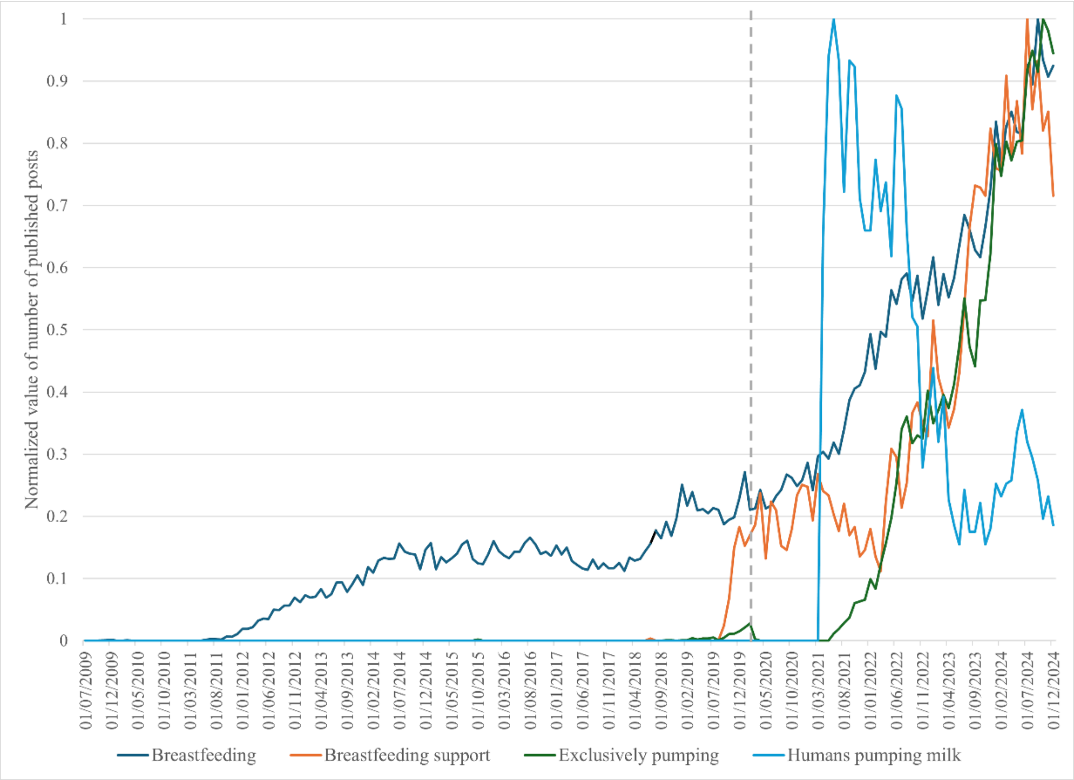


**Supplementary Figure 2.** Monthly counts of submitted posts to the subreddits *r/breastfeeding*, *r/breastfeedingsupport*, *r/ExclusivelyPumping*, and *r/HumansPumpingMilk* from their respective onsets through December 2024

**Legend.** For comparability across communities of different sizes and lifespans, submission counts were normalized to a 0-1 scale within each subreddit, with higher values indicating periods of relatively increased posting activity. Subreddits differ in inception dates; absence of activity reflects non-existence rather than zero posting. The grey dashed line indicates the onset of the COVID-19 pandemic (March, 2020). An overall growth trajectory is observed for most subreddits, apart from *r/HumansPumpingMilk*. Both long-term growth trends and short-term fluctuations in community engagement are evident, with particularly marked increases in activity for pumping-related subreddits after 2020.

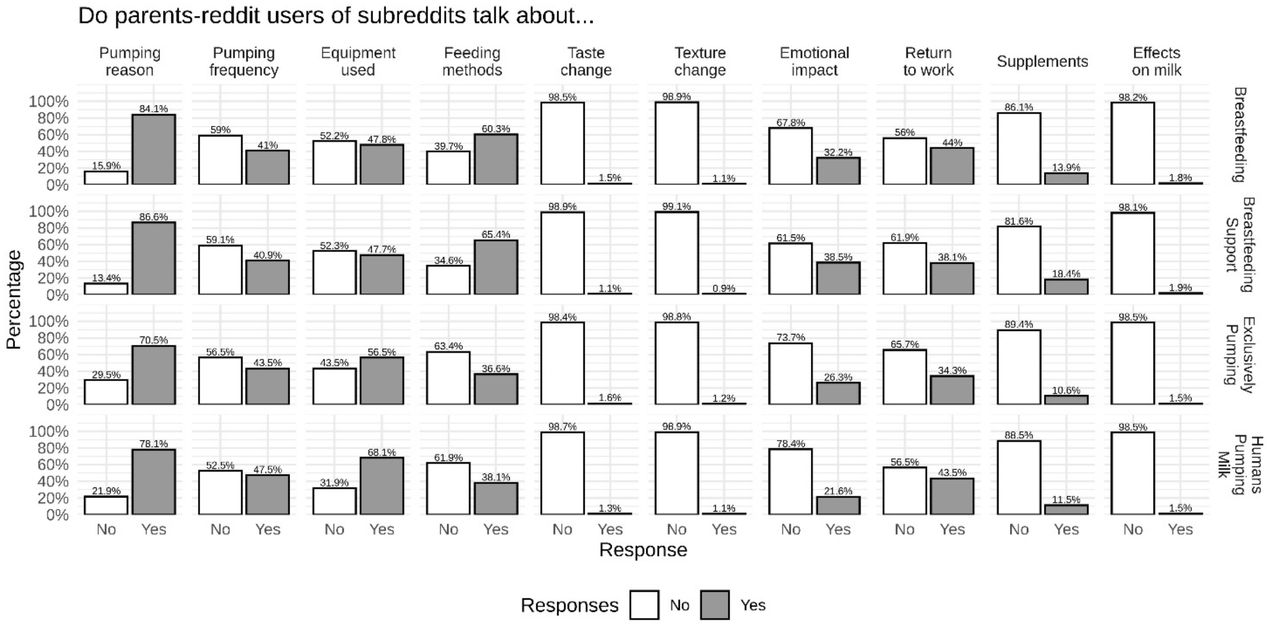


**Supplementary Figure 3.** Distribution of topic mentions across pumping-related discussion categories by subreddit

**Legend.** Overall, patterns of topic mention were broadly comparable across subreddits, with practical aspects of pumping dominating discussions in all communities. Pumping-focused subreddits showed higher frequencies of equipment-related discussions, whereas breastfeeding-oriented communities more often referenced feeding methods. Sensory concerns remained consistently rare across all subreddits, while emotional impacts and return-to-work considerations exhibited moderate variation, suggesting differences in the contextual framing of pumping experiences across communities.

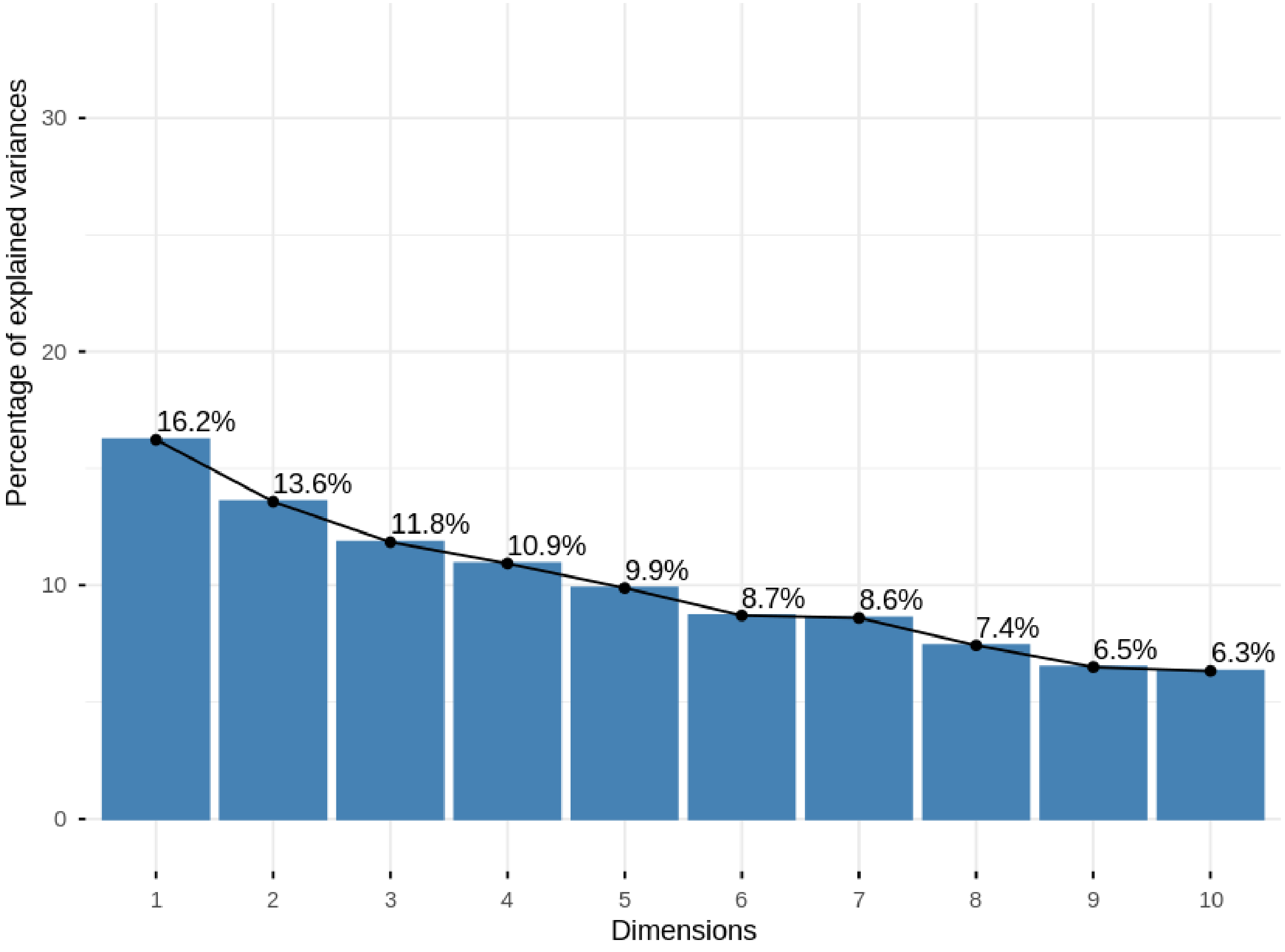


**Supplementary Figure 4.** Scree plot of multiple correspondence analysis dimensions

**Legend.** The scree plot displays the proportion of total inertia explained by each dimension derived from the multiple correspondence analysis (MCA) of the ten binary classification variables (shown in Table 2 of main text, with accuracy >75%). Dimensions are ordered according to decreasing explained inertia. The plot was used to assess the relative contribution of successive dimensions and to inform the selection of dimensions retained for interpretation.
